# Postoperative malocclusion after maxillofacial fracture management: a retrospective case study

**DOI:** 10.1186/s40902-018-0167-z

**Published:** 2018-10-15

**Authors:** Sang-Yun Kim, Yong-Hoon Choi, Young-Kyun Kim

**Affiliations:** 1 Department of Oral and Maxillofacial Surgery, Section of Dentistry, Seoul National University Bundang Hospital, 82 Gumi-ro, 173 Beon-gil, 300 Gumi-dong, Bundang-gu, Seongnam, 13620 South Korea; 2 Department of Dentistry, Dental Research Institute, School of Dentistry, Seoul National University, Daehak-ro 101, Jongno-gu, Seoul, 03080 South Korea; 3 Department of Conservative Dentistry, Section of Dentistry, Seoul National University Bundang Hospital, Seongnam, South Korea

**Keywords:** Maxillofacial fracture, Malunion, Complication

## Abstract

**Purpose:**

Various complications occur when a maxillofacial fracture is malunionized or improperly resolved. Malocclusion is the most common complication, followed by facial deformity, temporomandibular joint disorder (TMD), and neurological symptoms. The purpose of this study was to evaluate the dental treatment of postoperative complications after maxillofacial fracture.

**Materials and methods:**

In this study, nine patients with a postoperative complication after maxillofacial fracture who had been performed the initial operation from other units and were referred to the authors’ department had been included. Of the nine patients, six had mandibular fractures, one had maxillary fractures, one had maxillary and mandibular complex fractures, and one had multiple facial fractures. All the patients had tooth fractures, dislocations, displacements, and alveolar bone fractures at the time of trauma, but complications occurred because none of the patients underwent preoperative and postoperative dental treatment. Malocclusion and TMD are the most common complications, followed by dental problems (pulp necrosis, tooth extrusion, osteomyelitis, etc.) due to improper treatment of teeth and alveolar bone injuries. The patients were referred to the department of dentistry to undergo treatment for the complications. One of the nine patients underwent orthognathic surgery for a severe open bite. Another patient underwent bone reconstruction using an iliac bone graft and vestibuloplasty with extensive bone loss. The other patients, who complained of moderate occlusal abnormalities and TMDs such as mouth-opening limitation, underwent occlusal treatment by prosthodontic repair and temporomandibular joint treatment instead of surgery.

**Results:**

One patient who underwent orthognathic surgery had complete loss of open bite and TMD after surgery. One patient who underwent reconstruction using an iliac bone graft had a good healing process. Other patients were treated with splint, injection, and physical therapy for mouth-opening limitation and temporomandibular joint pain. After treatment, the TMDs were resolved, but the remaining occlusal abnormalities were resolved with prosthetic restoration.

**Conclusions:**

Considering the severity of malocclusion and TMJ symptom and the feasibillity of reoperation, nonsurgical methods such as orthodontic and prosthodontic treatments and splint therapy can be used to manage the dental and TMD complication after the trauma surgery. However, reoperation needs to be strongly considered for severe malocclusion and TMD problem.

**Electronic supplementary material:**

The online version of this article (10.1186/s40902-018-0167-z) contains supplementary material, which is available to authorized users.

## Background

Treatment of maxillofacial fractures can be classified into surgical and nonsurgical methods. Maxillofacial fractures and mandibular condylar fractures without occlusion and functional problems can be treated well with nonsurgical methods. In this case, the incidence of complications such as temporomandibular joint disorder can be minimized by avoiding the movement of the fractured segments by performing only the intermaxillary fixation and removing the inflammatory product and preventing fibrous adhesion through arthrocentesis surgery in the case of mandibular condyle fracture [1]. In addition, it is advisable to approach the fractures in children nonsurgically as much as possible to prevent facial bone growth disorder or damage of the tooth germ [2]. However, if functional problems occur, surgical treatment should be considered [3]. In cases of complex or comminuted fracture of the maxillofacial region, fracture with malocclusion or limitation of mouth opening, or fracture with visible deformation, an open reduction and internal fixation (ORIF) procedure was usually performed. Surgery usually improves the function and esthetics but is often associated with postoperative complications. Complications that may occur after maxillofacial fracture surgery can be divided into immediate and delayed complications. Immediate complications include airway compromise, bleeding, and loss of or damage to teeth or bone, and delayed complications may include nonunion, malunion, nerve injury, infections, temporomandibular joint (TMJ) problems, and disocclusion [4]. Complications may occur after any operations but must be identified and resolved once they occur. The purpose of this study was to evaluate the dental treatment of malunioned maxillofacial fractures.

## Main text

### Materials and methods

In this study, nine patients with a postoperative complication after maxillofacial fracture who had been performed with the initial operation from other units and were referred to the authors’ department had been included. Of the nine patients, six had mandibular fractures, one had a maxillary fracture, one had a maxillomandibular complex fracture, and one had a panfacial fracture. One of the six mandibular fractures occurred in the mandibular body; two, in the mandibular ramus; one, in the mandibular body and ramus; and two, in the mandibular body and condyle. The medical records and radiographs of the patients were used to investigate the treatment of complications. We will report some of these cases together. This study was conducted under the approval of the institutional review board (IRB) of Seoul National University Bundang Hospital (No. B-1802-453-106).

#### Case 1

**Fig. 1:**
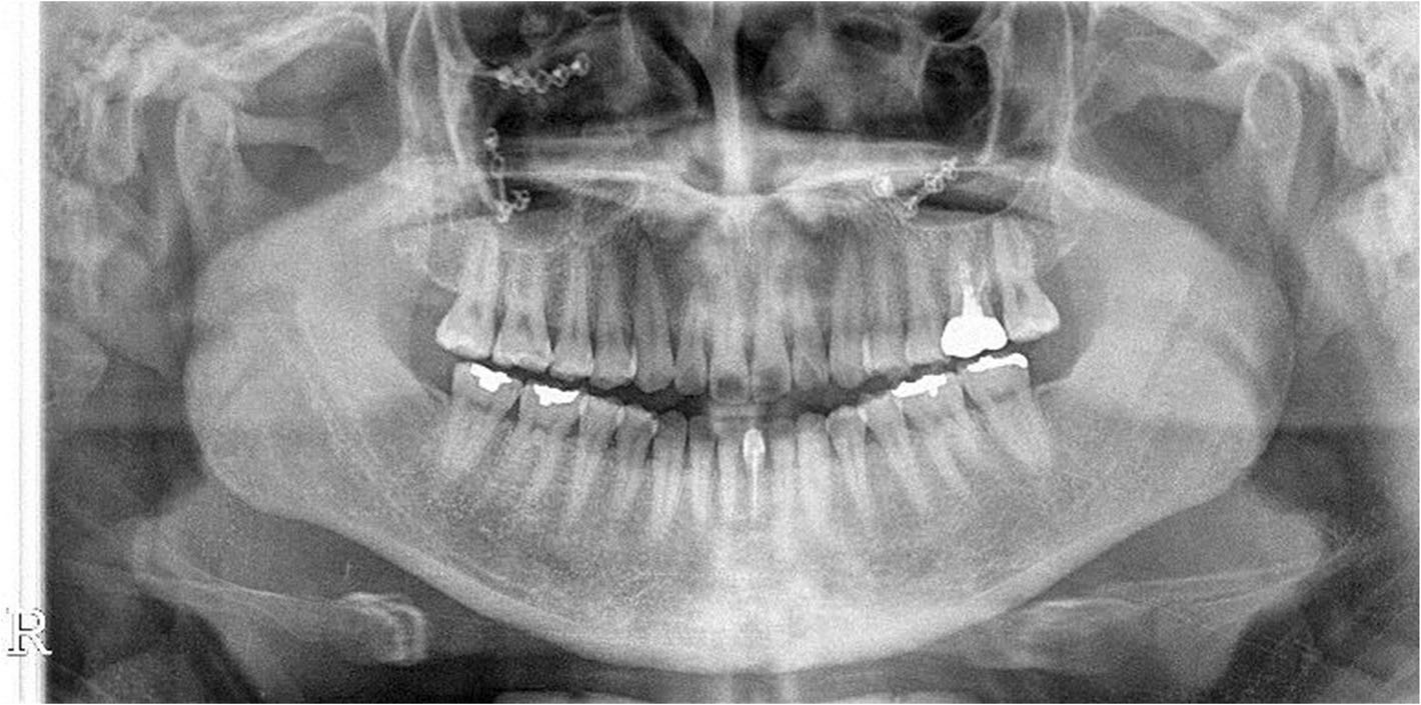
Preoperative panoramic radiograph showing malunioned segments both at the maxilla and severe malocclusion

**Fig. 2:**
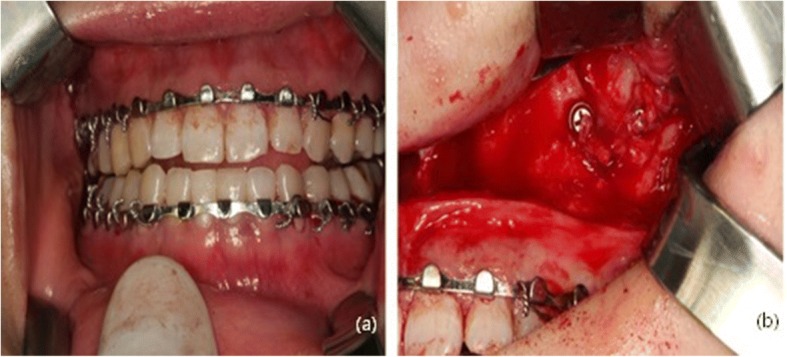
Clinical photograph during operation. **a** Severe malocclusion. Before reoperation, an arch bar was applied for intermaxillary fixation. **b** Upon exposure of the fractured site, malunioned segments with plate and screws were detected

**Fig. 3:**
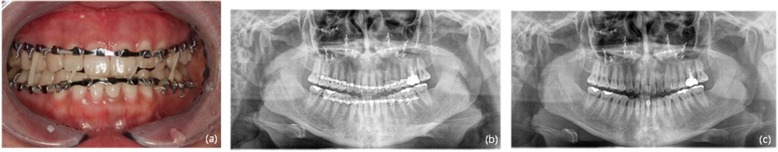
**a** Clinical photograph at 1 month after operation showing resolution of the malocclusion. **b** Panoramic radiograph at 1 month after operation. **c** Panoramic radiograph at 1 year after the operation

**Fig. 4:**
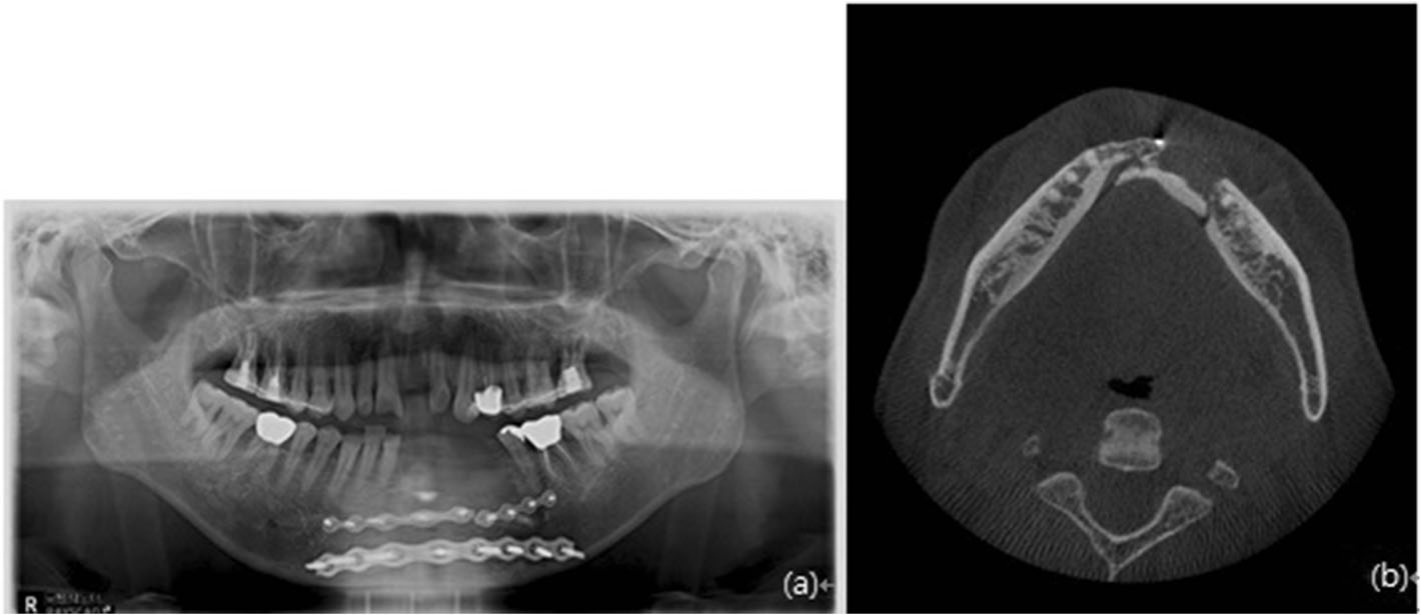
Preoperative radiograph. **a** Panoramic radiograph and **b** conical beam computed tomography scan

**Fig. 5:**
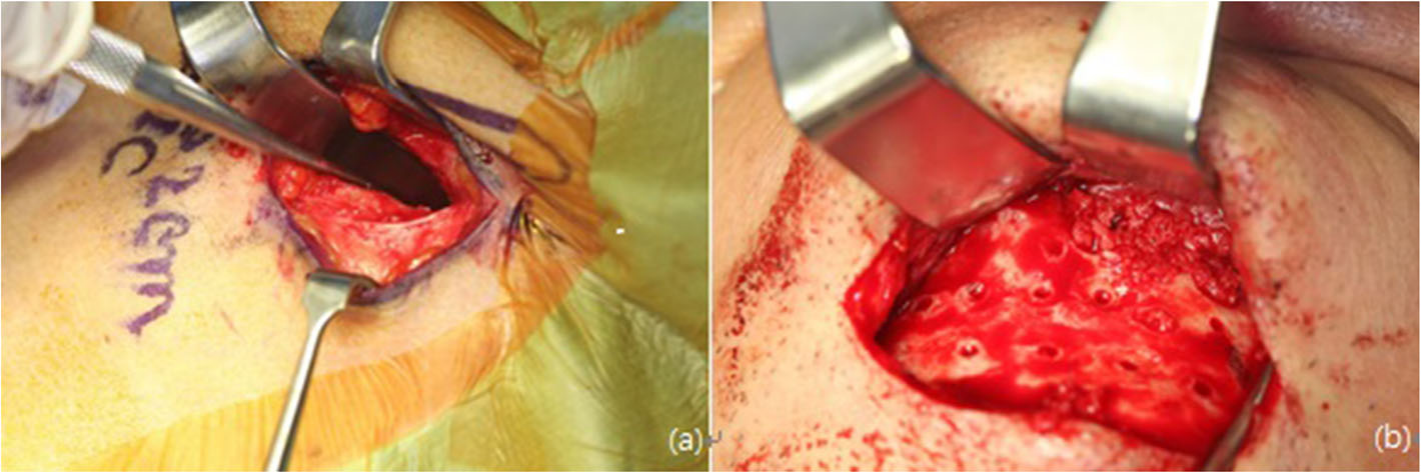
Intraoperative clinical photograph. **a** Harvesting of the iliac bone. **b** Grafting of the iliac bone at the mandibular defect area

A 29-year-old woman visited the Oral and Maxillofacial Surgery Department of Seoul National University Hospital in December 2014 to resolve a malocclusion after fracture surgery. In March 2014, she sustained a maxillary comminuted fracture from a traffic accident, and ORIF was performed in another hospital. Thereafter, she received splint treatment for severe malocclusion and TMJ pain. The patient was admitted to our clinic because of persistent severe malocclusion. We observed a 3-mm deviation to the left, a downward displacement of the maxillary on the right side, and severe open bite in which all teeth were not touching except for the upper and lower right second molars. In addition, the patient complained of severe pain in the right TMJ and face. Treatment with orthognathic surgery using maxillary Le Fort 1 osteotomy was finalized as a treatment plan for the unresolved malocclusion by orthodontic treatment alone (Figs. 1 and 2). After 1 month of orthognathic surgery, the patient was maintained in intermaxillary fixation. After that, the malocclusion was resolved, and the temporomandibular joint and facial tenderness disappeared and the treatment was terminated (Figs. 3, 4, and 5).

#### Case 2

**Fig. 6:**
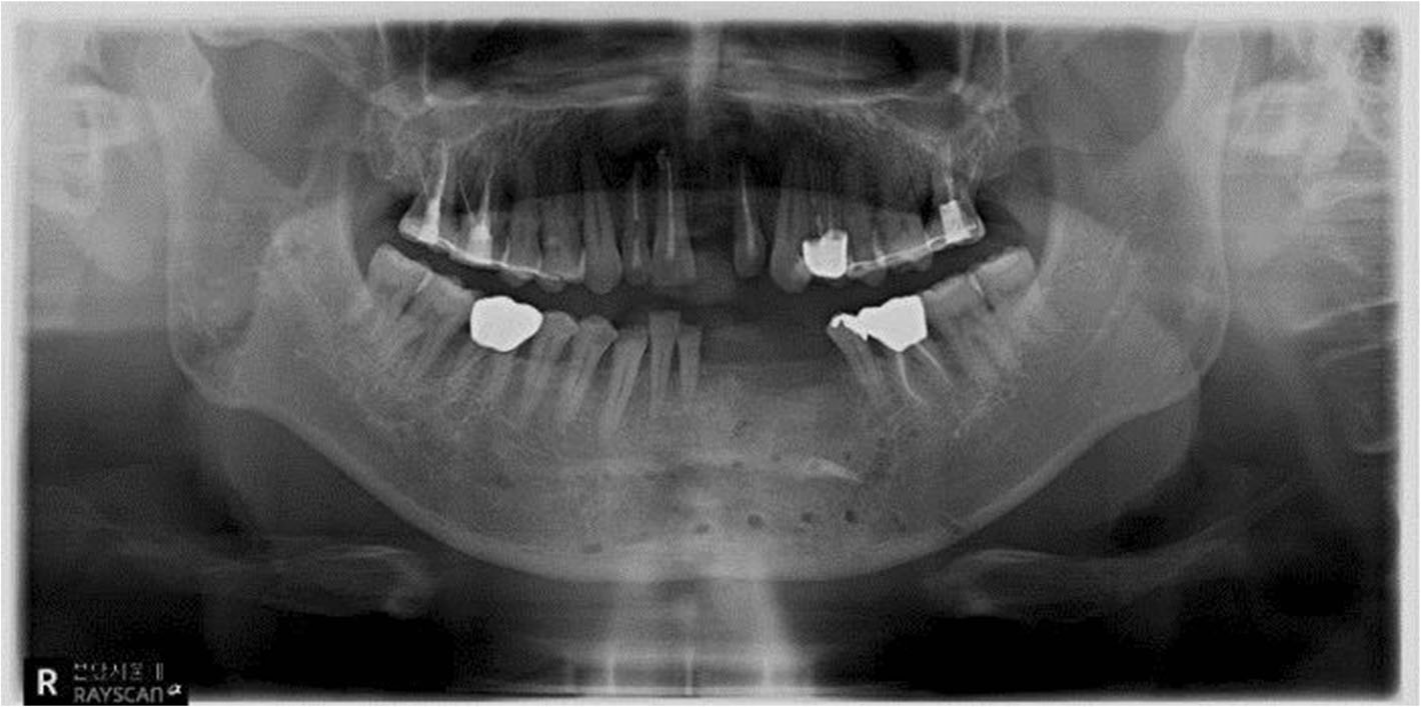
Postoperative panoramic radiograph

**Fig. 7:**
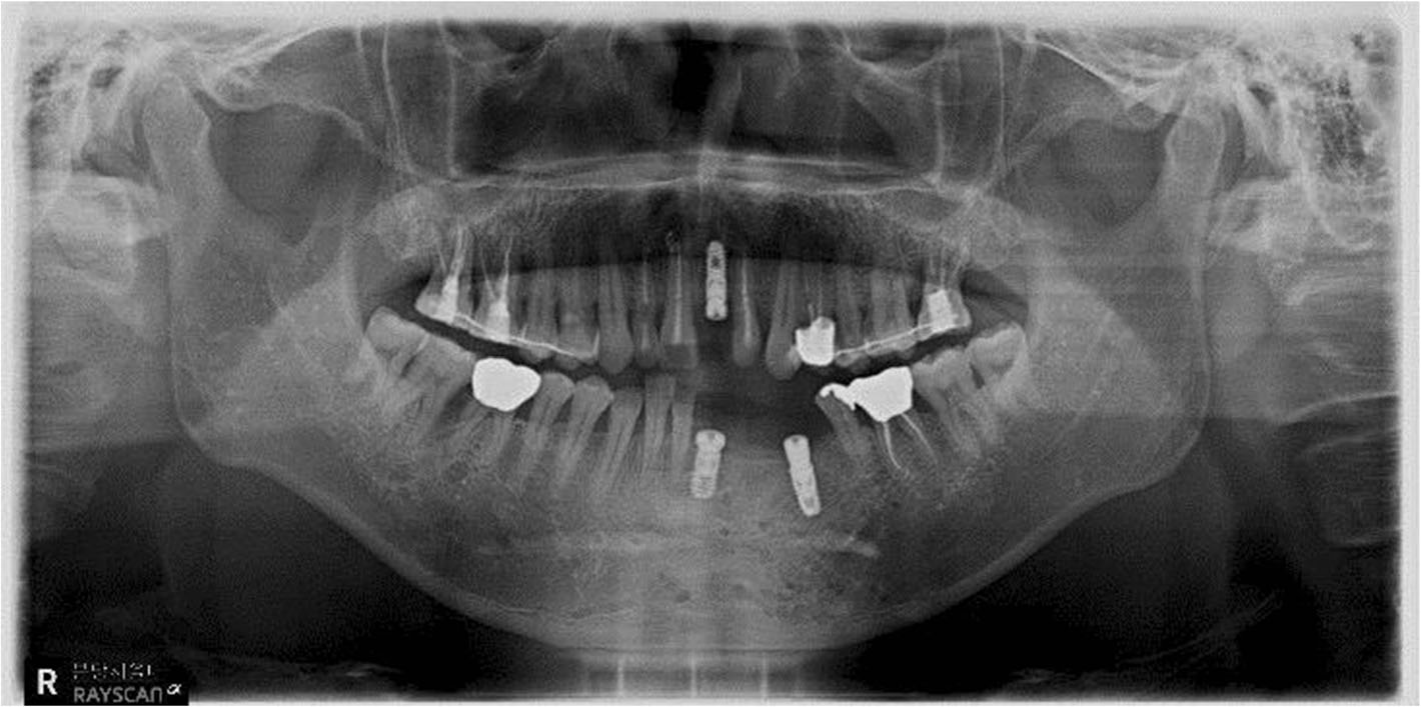
Panoramic radiograph after implant placement

A 54-year-old man underwent placement of several localized flaps for complicated bone exposures after ORIF surgery in another department because of an extensive fracture of the mandible in November 2016. Several teeth were removed without a plan during surgery. To solve the resulting dental problems, he was referred for oral and maxillofacial surgery. On examination, the maxillary left central incisor and mandibular left central incisors to the first premolar had disappeared. The incomplete fixation of the fractured segment of the anterior teeth area resulted in bone resorption. After the operation, the height of the vestibule was significantly decreased, resulting in an abnormal shape and movement of the lower lip and incomplete pronunciation. Computed tomography (CT) was performed to determine the exact state of the bone fragments and confirmed that the bone fragments at the fracture site were extensively resorbed owing to bony necrosis. Mandibular reconstruction for the removal of the misplaced metal plate and extensive mandibular bone loss, additional vestibuloplasty and scar revision, and subsequent implant placement were planned. Therefore, conservative treatment was performed for the teeth with pulp necrosis due to trauma until the operation. Finally, mandibular reconstruction using an iliac bone graft was planned. In May 2017, conventional metal plate removal and mandibular reconstruction using an iliac bone graft were performed under general anesthesia. Bone grafting was performed using the extracted ilium, alveolar bone fragments, and synthetic bone. Six months after the operation, adequate union of the bone fragment was observed on CT, and the implant was placed in the edentulous area. The primary stability of the implants was excellent, and the implants were implanted using a one-step procedure. An artificial dermis graft was performed on the soft tissue defects. Three months after the implantation, the prosthetic treatment was performed. Currently, the prosthesis is attached, and vestibuloplasty is planned (Figs. 6 and 7).

### Results

One patient (case 1) with extensive fractures in the maxilla was referred to our department because of severe malocclusion and facial asymmetry after the operation. The patient complained of severe open bite, severe deviation of the maxilla, and tenderness on the TMJ and face. The patient underwent a Le Fort I osteotomy under general anesthesia to resolve the malunion, and the malocclusion and TMD disappeared completely thereafter.

In one patient (case 2) with an extensive fracture of the mandible, multiple teeth were lost during an operation and postoperative complications resulted in extensive mandibular osteonecrosis, soft tissue loss, and malocclusion due to erroneous fixation of the fracture site. In this case, reconstruction using iliac grafting of the mandibular osteonecrosis site, implant restoration, and vestibuloplasty was planned for oral and maxillofacial surgery. After the removal of all necrotic bone fragments, reconstruction using the iliac and intraoral autogenous bones was performed under general anesthesia. After 6 months, implant placement and prosthetic treatment were completed.

In one case (case 3) of fracture in both the mandibular ramus and body, the patient complained of fracture and mobility of multiple teeth at the time of trauma and malocclusion due to the fracture. However, postoperative complications such as occlusal abnormalities, TMD, mouth-opening limitation, and pulp necrosis of the teeth occurred owing to the lack of proper dental treatment before and after the operation. Thereafter, the extraction of multiple teeth, implantation, endodontic treatment, prosthodontic restoration, and long-term treatment of TMJ were performed at the dental clinic.

One of the two patients (cases 4 and 8) who had a fracture near the mandibular ramus had a comminuted fracture at the left mandibular ramus and fracture and mobility of multiple teeth. At that time, the patient complained of occlusal abnormalities and toothache, but owing to the lack of proper dental treatment before and after surgery, he was referred to our dental clinic. Thereafter, several teeth were extracted, and restoration treatment proceeded. In one patient, fracture due to trauma occurred in the left mandibular ramus. After the operation at another department, mouth-opening limitation, TMJ pain, and sensory disturbance in multiple teeth occurred but were left untreated. Then, pus and edema occurred at the operation site but were left untreated until osteomyelitis developed. After the reoperation, the patient was referred to our dental clinic for further treatment.

In one patient with panfacial fractures (case 5), the fractures occurred in the bilateral orbital, maxillary, mandibular, and nasal bones during trauma, with fractures and dislocations of multiple teeth and extensive alveolar fractures. The operation was performed without proper dental treatment before the operation, and then the patient was referred to our dental clinic, but the systemic condition of the patient was severe and only the emergency treatment was completed.

One patient (case 6) with combined maxillary and mandibular fractures had comminuted fracture of the maxilla, fracture of the mandibular body and condyle, avulsion and dislocation of multiple teeth, and extensive fracture of alveolar bones. The dislocated teeth and fractured alveolar bone were not treated before and after the operation, and the displaced condyle was left untreated, resulting in postoperative complications such as loss of many teeth and TMD with mouth-opening limitation. The patient was referred to our dental clinic, where extensive bone grafting, implant placement, prosthodontic restoration, and prolonged temporomandibular joint treatment were performed.

One patient (case 7) who had fractures in the mandibular body and condyle had postoperative complications such as malocclusion and toothache due to condylar fixation without proper reduction, and tooth fractures and subluxation that occurred during trauma were left untreated at the time of surgery. The patient was referred to our dental clinic to undergo repair of the many teeth by using prosthodontic treatment.

In one case (case 9), fracture occurred in the mandibular body and both condyles. In the operation, only the mandibular body was reduced and both condyles were left untreated, resulting in malocclusion and temporomandibular disorders after the surgery. Thereafter, the patient was referred to our dental clinic for long-term treatment of the temporomandibular joints and teeth.

In summary, all the patients had fractures in the maxillofacial region accompanied by tooth fracture, dislocation, displacement, and alveolar bone fracture at the time of trauma. However, only ORIF of the fractured bone was performed, without preoperative and postoperative dental treatments. Malocclusion and TMD were the most common postoperative complications, and dental symptoms (dental necrosis, extrusion, osteomyelitis, etc.) due to improper treatment of teeth and alveolar bone injuries were frequent. All the patients were referred to our dental clinic to undergo treatment for the complications. One of the nine patients with severe open bite underwent orthognathic surgery. One patient with an extensive bone loss on the mandible underwent reconstruction using an iliac bone graft and vestibuloplasty. The other patients complained of moderate malocclusion and TMDs such as mouth-opening limitation and underwent occlusal treatment with prosthodontic restoration and temporomandibular joint treatment instead of surgical correction (Table 1).

**Table 1:** Patients’ information

Case	Sex	Age (years)	Injured site	Primary treatment	Postoperative complication	Final treatment
1	F	29	Mx. fx.	ORIF	Openbite (#17–47 contact only), maxillary deviation (malunion), TMD	Orthognathic surgery (Le Fort I osteotomy)
2	M	54	Mn. (body) and Mx. fx., multiple tooth luxation, and loss	ORIF, improper tooth extraction	Bone necrosis, malocclusion, tooth loss (#21, 31–34), tooth pain	Reconstruction with iliac bone graft, dental implantation, vestibuloplasty, scar revision
3	F	41	Mn. (body and ramus) fx., multiple tooth luxation, tooth fx.	ORIF without tooth reduction	Tooth pain, pulp necrosis, malocclusion, TMD	Dental tx. (tooth extraction, endodontic and prosthodontic tx.), TMD tx
4	M	24	Mn. (ramus) fx., tooth fx.	ORIF	Tooth problem	Dental tx. (tooth extraction, prosthodontic tx., implant)
5	M	18	Panfacial fx., multiple tooth luxation, tooth fx., alveolar bone fx.	ORIF without tooth reduction	Tooth loss, tooth mobility, alveolar bone resorption, malocclusion	Dental tx. (tooth extraction, endodontic and prosthodontic tx.)
6	F	37	Mx. and Mn. fx., tooth loss, multiple tooth luxation, tooth fx.	ORIF without tooth reduction	Condyle displacement, TMD, tooth loss, tooth pain, malocclusion	Dental tx. (tooth extraction, endodontic and prosthodontic tx.), TMD tx.
7	M	53	Mn. (body and condyle) fx., tooth fx. Tooth luxation	ORIF without tooth reduction	Tooth problem, malocclusion, TMD	Dental tx. (endodontic and prosthodontic tx.), TMD tx.
8	M	53	Mn. (ramus) fx. tooth luxation	ORIF, improper tooth extraction	Malocclusion, TMD, and osteomyelitis	Saucerization, TMD tx.
9	F	56	Mn. (body and both condyle) fx.	ORIF without condyle reduction	Tooth problem, malocclusion, TMD	Dental tx. (endodontic and prosthodontic tx.), TMD tx.

*TMD* temporomandibular disorder, *Mn* mandible, *Mx* maxilla, *tx* treatment, *fx* fracture, *ORIF* open reduction and internal fixation

### Discussion

Fractures of the maxillofacial region are often caused by traffic accidents, sports, or trauma. Singaram and Udhayakumar reported the following cases of maxillofacial fractures in developing countries. Fractures of the maxillofacial region caused by two-wheeled motor vehicles are the most frequent, especially in young men aged 20 to 40 years. Trauma is most common in the zygoma and maxilla, followed by the mandible. In the case of zygoma fractures, conservative treatment is often used, but maxillary and mandibular fractures often require reduction surgery [5].

Various complications can occur after fracture surgery of the maxillofacial region. Typically, these include tooth problems, soft tissue problems, nonunion, malunion/malocclusions, facial asymmetry, temporomandibular joint problems, nerve injury, osteonecrosis, and infection [6].

Tooth problems such as pulp necrosis, tooth fracture, and tooth dislocation can occur during surgery and may progress gradually over time. The most important strategy to prevent such problems is to perform initial emergency treatment immediately. Dental emergency treatment such as a resin-wire splint, pulp capping, pulpotomy, and pulpectomy can reduce the incidence of complications that may occur during and after surgery. In addition, soft tissue problems such as decreased vestibular height during incision and suture and scarring of the intraoral and extraoral cavities can be prevented if the operation is performed with caution. If a problem arises, vestibuloplasty, soft tissue augmentation, and scar revision can be used to resolve the problem later. In the case of a fracture of the mandibular ramus or subcondyle, surgery is often performed using the extraoral approach. Owing to the characteristics of the extraoral approach, scarring may occur in the facial area, and the intraoral approach tends to be used as much as possible, but scarring can be reduced sufficiently through accurate incision and suture. Many papers have reported that access to the surgical site is easier, and the reduction of the fracture fragment is more accurate when operating with the extraoral approach [7].

Nonunion occurs when the postoperative healing process to achieve a union of the bone is stopped [8]. Haug and Schwimmer defined nonunion as the case of having mobility in the fracture site after 4 weeks without surgery or 8 weeks after surgery when the fracture occurred [9].

Nonunion occurs for many reasons, including soft tissue infections, osteomyelitis, mobility in fracture site, inaccurate reduction, delayed healing, teeth present in the fracture site, drug or alcohol abuse, the surgeon’s lack of medical skills, and inadequate patient conditions [10]. The diagnosis of nonunion can be clinically evaluated mainly on the basis of the mobility and tenderness of the fracture site. The presence of loosening at the distal end of the fracture line may also be helpful in the diagnosis if the radiopacity at the fracture site is irregular on radiographs. Once nonunion is identified, the maxillomandibular fixation (MMF) should be removed and closed reduction should be attempted.

Malunion is defined when bone union occurs while being inaccurately reduced between fractured segments. The most common symptom and a sign of malunion is malocclusion [11]. If malunion is found early, the fractured segments should be re-reduced and fixed, or the MMF should be loosened so that occlusion can be restored. If resolving to the original state is impossible, prosthetic restoration or orthodontic treatment is performed to resolve the malocclusion. However, this may result in damage to several teeth, financial/temporal problems, and TMDs.

Facial asymmetry is also a complication of fracture surgery. In the early postoperative period, it usually appears in the wrong healing state due to improper reduction. In the long term, it may appear as an extension line of the abovementioned nonunion, malunion, and malocclusion. Facial asymmetry is difficult to diagnose in the early postoperative period. When the clinical examination is not successful because of severe facial swelling after surgery, the medical staff should always check whether the fractured parts are correctly reduced, or whether infection and malocclusion are present on radiographs before and after surgery. When facial asymmetry occurs owing to incorrect reduction of the mandible, the possibility of TMD occurring in the long term because of improper positioning of the mandibular condyle should be considered [12].

If a fracture occurs in the maxillofacial region, incomplete reduction of the mandibular condyle may cause the soft tissue defects around the condyle (e.g., joint disk), potential growth disturbance, ankylosis of the condyle, and malocclusion. As a result, various temporomandibular joint problems occur. In the case of such a complication, the basic solution is to resolve the problems of reduction of the condyle to its original position and the inflammation and fibrosis of the soft tissue around the TMJ. However, if rehabilitation through reoperation is not feasible, secondary treatment of the TMJ must be performed. It is aimed to prevent joint problems such as mouth-opening limitation and pain by preventing fibrous adhesions and removing inflammation through TMJ arthrocentesis, injection, splinting, and physical therapy [13]. In addition, osteonecrosis or infection can occur. Osteonecrosis occurs mainly when healing is delayed owing to the improper blood supply to the fracture site, and infection may occur owing to inadequate antibiotic therapy and disinfection before and after surgery [14].

Among the fractures of the maxillofacial region, nerve injuries are common, especially when mandibular fractures occur. It is mainly associated with the inferior alveolar nerve, and it is more frequent in the mandibular ramus fractures than in the mandibular body fractures. Most nerve injuries are likely to recover if early appropriate treatment is applied but cannot be recovered if the damage by the fracture itself or by the wrong operation is irreversible [15]. An impacted third molar may cause nerve injury. Complications such as nerve injury, TMD, malocclusion, and infection have been reported in cases where a fully or incompletely impacted third molar is located in the fracture line, and the fractured segments are fixated without extraction during fracture surgery. Therefore, if the extraction of the third molar is possible, extraction during surgery should be considered [16].

Various complications can occur after fracture surgery. Postoperative complications cannot be prevented but can be minimized if a treatment plan is established through precise identification of the cause before surgery. In this study, we report cases of postoperative complications when only ORIF surgery was performed, without proper evaluation of occlusion, temporomandibular joint, tooth damage, and so on. Once a complication occurs, the cause must be identified to develop a solution. However, these cases were referred to our dental clinic without any treatment of the complications in the surgical field. Nevertheless, the dental complications were solved. As a result, the total treatment period was extended, and the treatment cost was increased.

## Conclusions

In cases of complications such as malocclusion and temporomandibular joint disorders due to malunion, recovering the original state through reoperation is the first solution. However, if the severity of the complication is minimal and the problem is solved using nonsurgical methods, if reoperation is impossible because reopening would take a long time, or if the economic condition or aftereffects of surgery is expected to be greater, the complication can be solved using nonsurgical methods such as orthodontic, prosthodontic, and splint therapy. Thereby, a clinically acceptable state can be recovered.

## Additional file

Additional file 1: Case form and result of data. (XLSX 10 kb)

## Abbreviations

CT: Computed tomography
IRB: Institutional review board
MMF: Maxillomandibular fixation
Mn: Mandible
Mx: Maxilla
ORIF: Open reduction and internal fixation
TMD: Temporomandibular joint disorder
TMJ: Temporomandibular joint
tx: Treatment
